# Heterogeneous Oral Health Outcomes by Combustible Cigarette Smoking, E‐Cigarette Use and Dual Use: A Cross‐Sectional Population‐Based Analysis

**DOI:** 10.1111/odi.70390

**Published:** 2026-06-12

**Authors:** Yusuff Adebayo Adebisi, Najim Z. Alshahrani, Mohammed Moshabbab Alqahtani, Roqayya Mohammed Ahmed Alhayyani, Mona Majrashi, Anas Ali Alhur, Waleed Almutairi, Oshibe Joseph Daberechi, Isaac Olushola Ogunkola

**Affiliations:** ^1^ College of Social Sciences University of Glasgow Glasgow UK; ^2^ Nuffield Department of Population Health University of Oxford Oxford UK; ^3^ Department of Family and Community Medicine, Faculty of Medicine University of Jeddah Jeddah Saudi Arabia; ^4^ Department of Preventive Medicine Asir Cluster Abha Saudi Arabia; ^5^ Ministry of Health, Asir Cluster Abha Saudi Arabia; ^6^ Aseer Health Cluster Khamis Mushyt Saudi Arabia; ^7^ Department of Health Informatics, College of Public Health and Health Informatics University of Hail Hail Saudi Arabia; ^8^ King Fahad Military Medical Complex Dhahran Saudi Arabia; ^9^ Medical Laboratory Science Department Ebonyi State University Abakaliki Nigeria; ^10^ Bristol Medical School University of Bristol Bristol UK

**Keywords:** dual use, e‐cigarettes, gum disease, OHIP‐14, oral health, population survey, self‐rated oral health, smoking

## Abstract

**Background:**

Combustible cigarette smoking is a well‐established risk factor for poor oral health, but the implications of e‐cigarette use and dual use remain uncertain. Distinguishing the effects of vaping from the lingering consequences of prior smoking is a persistent challenge in the literature.

**Objectives:**

To assess how distinct nicotine use profiles relate to oral health–related quality of life, self‐reported oral health indicators and self‐rated dental health among adults in England, while differentiating e‐cigarette users by smoking history.

**Methods:**

We conducted a cross‐sectional analysis of 6027 adults (aged ≥ 16 years) from the nationally representative 2021 Adult Oral Health Survey for England. Participants were classified into five current smoking/vaping categories (never users, former smokers, exclusive cigarette smokers, exclusive e‐cigarette users, dual users) and, separately, by combined vaping–smoking history. Outcomes included any oral health difficulty and the number of impacts derived from the 14‐item Oral Health Impact Profile (OHIP‐14), as well as loose teeth, gum disease history, dry mouth and self‐rated dental health. Binary outcomes (any oral health difficulty, loose teeth, gum disease history) were analysed using logistic regression, and ordinal outcomes (number of OHIP‐14 impacts, dry mouth, self‐rated dental health) using ordered logistic regression, with adjustment for sociodemographic, behavioural and oral hygiene covariates.

**Results:**

Compared with never users, exclusive cigarette smokers had the highest adjusted odds of reporting any oral health difficulty (OR = 1.85, 95% CI: 1.51–2.27), loose teeth (OR = 3.18, 95% CI: 2.47–4.09), gum disease history (OR = 1.94, 95% CI: 1.52–2.49) and poorer self‐rated dental health (OR = 2.88, 95% CI: 2.36–3.52). Dual users showed similarly elevated odds for loose teeth (OR = 3.11, 95% CI: 1.95–4.97), the highest odds for gum disease history (OR = 3.21, 95% CI: 2.06–4.99) and elevated odds for any oral health difficulty (OR = 1.55, 95% CI: 1.04–2.30) and poorer self‐rated dental health (OR = 2.45, 95% CI: 1.68–3.58). Exclusive e‐cigarette users also showed elevated odds across outcomes, though generally of smaller magnitude. Estimates for the very small subgroup of never‐smoking current e‐cigarette users did not show significant excess odds across outcomes, but were imprecise and should be regarded as exploratory.

**Conclusion:**

In this cross‐sectional population‐based analysis, combustible cigarette smoking and dual use were associated with poorer self‐reported oral health outcomes. Findings related to e‐cigarette use require cautious interpretation because of prior smoking history, the sparse never‐smoker vaping subgroup and the absence of detailed exposure data (e.g., smoking intensity, pack‐years, time since cessation, vaping duration, device type and nicotine concentration). Smoking cessation should remain central to preventive dentistry.

## 1 Introduction

Oral health is a core component of general well‐being, yet it remains widely compromised by preventable risk factors, most notably the use of nicotine and tobacco products (Gajendra et al. 2023; Chaffee et al. 2022, 2021). The detrimental effects of combustible cigarette smoking on oral tissues are well established, including increased risks of periodontal disease, impaired healing, soft‐tissue damage and altered salivary function (Tomar et al. 2019; Silveira et al. 2022). These biological consequences often translate into poorer oral health‐related quality of life, characterised by pain, reduced ability to eat and psychosocial disturbances such as embarrassment and diminished life satisfaction (Hassan et al. 2025; Sagtani et al. 2020). Despite sustained public health investment in smoking cessation, nicotine use remains prevalent and continues to evolve in ways that complicate oral health policy and clinical practice.

In recent years, electronic cigarettes have transformed patterns of nicotine consumption (Baldassarri 2020; Jackson, Cox, et al. 2025b; Jackson, Brown, et al. 2025a). Frequently marketed as a safer alternative to smoking, e‐cigarette use (vaping) has been widely adopted among current and former smokers, as well as among individuals who have never smoked combustible cigarettes (Jackson, Cox, et al. 2025b). Although e‐cigarettes eliminate combustion products, concerns include potential alterations to soft‐tissue responses, salivary function, inflammatory processes and the oral microbiome (Cichońska et al. 2024; Szumilas et al. 2022; Yang et al. 2025). The co‐use of cigarettes and e‐cigarettes, commonly referred to as dual use, introduces further ambiguity, as the combined exposure may intensify risk rather than reduce it. These contrasting narratives generate uncertainty for clinicians, policymakers and users as to whether e‐cigarette use represents a harm reduction pathway or an additional source of oral health burden.

A persistent challenge in the literature is separating the impact of e‐cigarette use itself from the lingering consequences of past smoking (Polosa et al. 2025; Hajat et al. 2022; Spicuzza et al. 2025). Many studies classify all e‐cigarette users together, even though most are former smokers, which risks attributing smoking‐related effects to e‐cigarette use (Polosa et al. 2025; Hajat et al. 2022; Spicuzza et al. 2025). Robust national data examining distinct smoking and e‐cigarette behaviour patterns alongside patient‐reported oral health outcomes are scarce (Iacob et al. 2024; AlQobaly et al. 2022; Robson et al. 2025). Addressing these gaps is essential for improving scientific understanding and guiding dental prevention strategies.

Accordingly, this study draws on nationally representative data to assess how distinct nicotine use profiles relate to oral health among adults in England. First, we examined differences in oral health–related quality of life using the full set of Oral Health Impact Profile (OHIP) items, summarised descriptively and operationalised through both a composite indicator of any reported difficulty and an ordinal measure capturing the accumulation of impairments. Second, we estimated the associations between smoking and e‐cigarette status and three self‐reported oral health indicators: loose teeth, gum disease history and dry mouth. Third, recognising the importance of exposure history, we differentiated current e‐cigarette users into those who had previously smoked and those who had never smoked, enabling clearer interpretation of whether observed effects reflect e‐cigarette use itself or residual consequences of past smoking. Finally, to capture broader perceptions of oral health, we assessed self‐rated dental health using a validated five‐category question and modelled its association with nicotine use patterns, providing an additional patient‐centred indicator of perceived oral wellbeing.

## 2 Data Source, Study Design and Participants

This study used data from the 2021 Adult Oral Health Survey (AOHS), a nationally representative survey commissioned by Public Health England and delivered by NatCen Social Research in partnership with academic collaborators from the University of Birmingham, King's College London, Newcastle University, University College London and the Office for National Statistics (Public Health England 2021). The AOHS is conducted approximately every 10 years to monitor trends in oral health across England and inform National Health Service planning (Public Health England 2021). A stratified random sample of household addresses was selected from the national postcode file maintained by the Office for National Statistics, and all adults aged 16 years and over residing at sampled addresses were invited to take part (NatCen Social Research 2022). Survey materials were distributed via post, directing eligible participants to an online questionnaire that collected detailed information on oral health behaviours, symptoms and impacts, dental attendance, smoking and vaping behaviours and general health. Participation was voluntary, and responses were anonymised in accordance with UK General Data Protection Regulation. Ethical approval for the AOHS was granted by NatCen's independent Research Ethics Committee (Public Health England 2021; NatCen Social Research 2022). A total of 6321 adults participated in the 2021 survey. For the present analysis, we excluded respondents with missing data on smoking status, vaping status, oral health outcomes or adjustment variables (*n* = 294). After these exclusions, 6027 adults were retained in the analytic sample.

## 3 Exposure Assessment

Two complementary exposure schemes were used throughout the analysis. The primary scheme (five‐category current smoking/vaping status) captured present nicotine use behaviours, comprising never users, former smokers (not current vapers), exclusive cigarette smokers, exclusive e‐cigarette users and dual users. The secondary scheme (four‐category combined vaping–smoking history) was designed to disentangle e‐cigarette use from prior smoking exposure, comprising never e‐cigarette users, former e‐cigarette users, current e‐cigarette users who had never smoked and current e‐cigarette users with a smoking history. Both schemes were derived from the same source variables (SmkDV and VapDV) and are described in detail below. The two schemes are referenced consistently across all subsequent analyses, with each results table indicating which scheme was applied.

Cigarette smoking was measured using the variable SmkDV, which classified respondents into three categories based on their self‐reported cigarette use: current smokers, former smokers and never smokers. E‐cigarette use was measured using the variable VapDV, which similarly classified respondents as current e‐cigarette users, former e‐cigarette users or never users of e‐cigarettes or vaping devices.

### 3.1 Primary Exposure: Current Smoking–Vaping Status

For the main analyses, SmkDV and VapDV were combined to create a five‐category exposure variable representing current smoking and vaping behaviour. Participants were classified as:

1. *Never users:* never smoked cigarettes and never used e‐cigarettes.
2. *Former smokers, not current vapers:* previously smoked cigarettes but do not currently smoke or vape; this group includes individuals who have never vaped and those who only vaped in the past.
3. *Current exclusive cigarette smokers:* currently smoke cigarettes but do not currently vape, regardless of past e‐cigarette use.
4. *Current exclusive e‐cigarette users:* currently vape but do not currently smoke; this category includes both ex‐smokers who now vape exclusively and the small number of never smokers who vape.
5. *Current dual users:* currently smoke cigarettes and currently use e‐cigarettes.

This variable captures current nicotine exposure pathways, allowing comparisons between exclusive smoking, exclusive vaping, dual use and non‐use.

### 3.2 Secondary Exposure: Vaping–Smoking History

To investigate behavioural history and patterns of uptake, a second exposure variable was derived that jointly classified respondents according to past and current e‐cigarette use behaviour and their smoking history. This yielded four categories:

1. *Never e‐cigarette users:* never used e‐cigarettes; this group includes current smokers, former smokers and never‐smokers.
2. *Former e‐cigarette users:* formerly used e‐cigarettes but are not current users; in this dataset all former vapers had a history of cigarette smoking.
3. *Current e‐cigarette users—never smokers:* currently use e‐cigarettes but have never smoked cigarettes.
4. *Current e‐cigarette users—ever smokers:* currently vape and have a history of cigarette smoking (either former or current smokers).

This variable captures behavioural trajectories into vaping, distinguishing long‐term patterns (e.g., former e‐cigarette users) and separating exclusive e‐cigarette users into those who have and have not smoked.

## 4 Outcome Measures

This study examined five oral health outcomes (operationalised through six analytic measures, as the OHIP‐14 was assessed using both a binary ‘any difficulty’ indicator and an ordinal count of impacts) derived from the adult dental health module. These outcomes captured both self‐reported clinical indicators (loose teeth and gum disease) and subjective assessments of oral health experience and burden, including oral health–related quality of life, accumulated functional impacts and self‐rated dental health.

### 4.1 Oral Health‐Related Quality of Life

Two outcomes were derived from the 14‐item Oral Health Impact Profile (OHIP‐14), which asked participants how often, in the last 12 months, problems with their teeth, mouth or dentures had affected them. Each item offered five response categories: *Never*, *Hardly ever*, *Occasionally*, *Fairly often* and *Very often*. The 14 items covered functional limitation, physical pain, psychological discomfort, physical disability, psychological disability, social disability and handicap (i.e., trouble pronouncing any words; feeling that sense of taste has worsened; painful aching in the mouth; discomfort when eating foods; being self‐conscious; feeling tense; diet being unsatisfactory; having to interrupt meals; finding it difficult to relax; being a bit embarrassed; being irritable with other people; difficulty doing usual jobs; life feeling less satisfying or being totally unable to function).

#### 4.1.1 Any Oral Health Difficulty (Binary)

For each item, responses of *Occasionally*, *Fairly often* or *Very often* were coded as indicating difficulty, while *Never* and *Hardly ever* were coded as no difficulty. The composite outcome was coded as 1 if the participant reported difficulty on at least one OHIP item and 0 otherwise. This measure captures the presence of any negative oral health impact.

#### 4.1.2 Number of Oral Health Impacts (Ordinal)

To quantify overall burden, the number of OHIP‐14 items endorsed as difficulties was summed and recoded into a four‐level ordered variable: 0 = none; 1 = one item; 2 = two to four items and 3 = five or more items. This ordinal score represents a graded measure of increasing severity and frequency of functional, psychological and social impacts related to oral health.

### 4.2 Loose Teeth

Loose teeth were assessed using the question: ‘Have you ever had any teeth become loose on their own, without an injury?’ with response options *Yes* and *No*. The binary variable was coded as 1 for ‘Yes’ and 0 for ‘No’.

### 4.3 Gum Disease History

Gum disease history was identified using the question: ‘Have you ever been told by a dental health professional (for example a dentist or hygienist) that you have gum disease? This may be described as having deep pockets or losing bone around the teeth’. Participants answered *Yes* or *No*. The binary variable was coded as 1 for ‘Yes’ and 0 for ‘No’.

### 4.4 Dry Mouth

Dry mouth was assessed with the question: ‘How often does your mouth feel dry?’ with four ordered categories: *Never*, *Occasionally*, *Frequently* and *Always*. The variable was treated as an ordered categorical outcome, with higher values indicating more frequent experiences of dry mouth.

### 4.5 Self‐Reported Dental Health

Self‐perceived dental health was measured using the question: ‘How is the health of your mouth in general?’ Participants selected one of five options: Very good, Good, Fair, Bad or Very bad. For analysis, responses were grouped into three ordered categories reflecting increasing levels of perceived oral health burden: Very good/good, Fair and Bad/very bad. This ordinal measure captures participants' overarching appraisal of their oral health status and has previously been used as a global indicator of lived oral health experience (Mariño et al. 2008; Atala‐Acevedo et al. 2023; Bond et al. 2024; Sekundo et al. 2019; de Jong et al. 2025).

## 5 Covariates

A set of sociodemographic, behavioural and oral hygiene variables were included as covariates based on theoretical relevance and previous evidence linking them to both nicotine use and oral health outcomes (Mbawalla et al. 2010; Lertpimonchai et al. 2017; Camila et al. 2025). Age group was categorised as 16–34, 35–54 and 55 years or older. Sex was coded as male or female. Ethnicity was dichotomised as White versus non‐White. Area deprivation was measured using national Index of Multiple Deprivation (IMD) quintiles ranging from most deprived to least deprived. Alcohol consumption was derived from AUDIT‐C scores and grouped as non‐drinker, low/medium‐risk drinking or heavy/dependent use. Ever use of smokeless tobacco was included as a binary measure (yes/no) due to its known oral effects. Oral hygiene and dental care behaviours were also adjusted for. Dental attendance was categorised into five levels: every 6 months, every year, every 2 years, less often than every 2 years or only when having problems. Fluoride toothpaste use was coded as yes or no. Tooth‐cleaning frequency was dichotomised as regular versus irregular brushing. Regular brushing was defined as brushing twice daily or more often (including twice daily and more than twice daily). Brushing less than twice daily was considered irregular. This categorisation aligns with clinical recommendations that twice‐daily brushing represents the minimum standard for optimal oral hygiene (NHS 2025).

## 6 Statistical Analysis

Descriptive statistics were first generated to characterise the analytic sample, with categorical variables summarised using frequencies and percentages. As an initial step, comparative oral health burden analyses were conducted. The prevalence of each OHIP‐14 impact was cross‐tabulated by current smoking/vaping status and by vaping‐smoking history to identify patterns in oral health‐related quality of life across exposure groups. These comparisons allowed descriptive examination of gradients in reported difficulty before inferential modelling.

Two exposure frameworks were analysed. First, a five‐category current smoking/vaping status variable compared never users, former smokers not currently using e‐cigarettes, exclusive cigarette smokers, exclusive e‐cigarette users and dual users. Second, a four‐category vaping‐smoking history variable distinguished never e‐cigarette users, former e‐cigarette users, current e‐cigarette users who had never smoked, and current e‐cigarette users with a history of smoking. For both frameworks, exposure categories were treated as nominal, with ‘Never users’ or ‘Never e‐cigarette users’ serving as the reference group.

Six outcome measures were assessed. Three binary outcomes, any oral health difficulty, loose teeth and gum disease history, were analysed using logistic regression. Three ordered outcomes, frequency of dry mouth, grouped number of OHIP impacts and self‐rated dental health, were analysed using ordered logistic regression. Self‐rated dental health was modelled as a three‐level ordinal outcome: very good/good, fair and bad/very bad. The proportional odds assumption was assessed for ordered logistic models using post‐estimation diagnostics and model comparisons; ordered logistic models were retained where no material violations were identified.

All primary adjusted models controlled for age group, sex, ethnicity, Index of Multiple Deprivation quintile, alcohol consumption, ever use of smokeless tobacco, fluoride toothpaste use, dental attendance frequency and brushing frequency. All covariates were modelled categorically. Odds ratios and 95% confidence intervals were reported for all models, with statistical significance defined as *p* < 0.05.

Sensitivity analyses were conducted by additionally adjusting the fully adjusted models for denture status, number of natural teeth and self‐rated general health. Denture status was modelled as any denture versus no denture. Number of natural teeth was modelled as 21 or more, 1–20 and no natural teeth. Self‐rated general health was modelled as very good/good, fair and bad/very bad. These variables were included in sensitivity rather than primary models because denture wearing and number of natural teeth may partly reflect accumulated oral disease and tooth loss, which could lie downstream of long‐term smoking exposure. For loose teeth and gum disease history models including dentition status, participants with no natural teeth were omitted where edentulous status perfectly predicted absence or non‐applicability of the outcome.

Because current e‐cigarette users who had never smoked formed a very small subgroup, Firth penalised logistic regression was conducted as a sparse‐data sensitivity analysis for the binary outcomes: any oral health difficulty, loose teeth and gum disease history. Model estimation was assessed using likelihood‐ratio tests, pseudo‐*R* ^2^ values, log‐likelihood statistics and convergence status. All primary, sensitivity and Firth penalised models converged successfully, and likelihood‐ratio tests confirmed that adjusted models provided a significantly better fit than crude models for all outcomes. All analyses were conducted using Stata 18.

## 7 Results

### 7.1 Sample Characteristics

Table 1 summarises characteristics of the 6027 adults included in the analysis. Almost half of participants were aged 55 years or older (46.9%), followed by those aged 35–54 years (34.3%) and 16–34 years (18.9%). Women comprised a larger proportion of the sample than men (57.4% vs. 42.6%). Most respondents identified as White (84.5%). Socioeconomic distribution across deprivation quintiles was relatively even, although the third (22.4%), fourth (24.2%) and least deprived quintiles (24.6%) were slightly over‐represented compared with the most deprived group (12.6%). Alcohol consumption was common, with 39.9% reporting low to medium risk drinking and 38.5% classified as heavy or dependent drinkers. A small proportion reported ever using smokeless tobacco (2.2%). Patterns of dental care and hygiene varied. Almost half attended dental services every 6 months (47.5%), whereas 11.4% sought care only when problems arose. Most participants reported regular toothbrushing (76.8%), and 77.0% reported using fluoride toothpaste. In terms of tobacco and nicotine exposure, 57.0% were classified as never users of cigarettes or e‐cigarettes, 30.7% were former smokers who were not currently vaping, 7.8% were exclusive cigarette smokers, 2.5% were exclusive e‐cigarette users and 1.9% were dual users. When vaping history was examined separately, the majority had never used an e‐cigarette (88.6%), 6.9% were former e‐cigarette users (all of whom had smoked), 0.2% were current e‐cigarette users who had never smoked and 4.2% were current e‐cigarette users with a smoking history.

**TABLE 1:** Descriptive characteristics of the study sample (*n* = 6027).

Characteristic	*n* (%)
Age group	
16–34	1137 (18.9)
35–54	2064 (34.3)
55+	2826 (46.9)
Sex	
Male	2569 (42.6)
Female	3458 (57.4)
Ethnicity	
White	5094 (84.5)
Non‐white	933 (15.5)
Index of Multiple Deprivation (IMD)	
Most deprived	757 (12.6)
Second quintile	981 (16.3)
Third quintile	1349 (22.4)
Fourth quintile	1456 (24.2)
Least deprived	1484 (24.6)
Alcohol consumption	
Non‐drinker	1306 (21.7)
Low/medium risk	2402 (39.9)
Heavy/dependent	2319 (38.5)
Ever smokeless tobacco use	
Yes	132 (2.2)
No	5895 (97.8)
Dental attendance	
Every 6 months	2863 (47.5)
Every year	1646 (27.3)
Every 2 years	463 (7.7)
Less frequent	370 (6.1)
Only when in trouble	685 (11.4)
Tooth‐cleaning frequency	
Regular brushing	4630 (76.8)
Irregular brushing	1397 (23.2)
Fluoride toothpaste use	
Yes	4639 (77.0)
No	1388 (23.0)
Current smoking/vaping status	
Never users	3436 (57.0)
Former smokers, not current e‐cigarette users	1852 (30.7)
Exclusive cigarette smokers	471 (7.8)
Exclusive e‐cigarette users	153 (2.5)
Dual users	115 (1.9)
Combined vaping–smoking history	
Never e‐cigarette users	5342 (88.6)
Former e‐cigarette users (all ever‐smokers)	417 (6.9)
Current e‐cigarette users—never smokers	13 (0.2)
Current e‐cigarette users—ever smokers	255 (4.2)

### 7.2 Comparative Oral Health Burden by Smoking and Vaping Status

Across OHIP items, never users generally had the lowest prevalence, former smokers were intermediate and exclusive cigarette smokers and dual users had the highest burden. Exclusive e‐cigarette users tended to show intermediate prevalences, often closer to former smokers, with the highest group varying by item (see Table 2).

**TABLE 2:** Prevalence of oral health impacts (OHIP‐14) by current smoking/vaping status.

OHIP item (any difficulty = yes)	Never users (%)	Former smokers (%)	Exclusive cigarette smokers (%)	Exclusive e‐cigarette users (%)	Dual users (%)
Had trouble pronouncing any words	3.7	5.2	9.8	5.9	9.6
Felt that your sense of taste has worsened	4.0	5.4	11.3	5.2	13.9
Had painful aching in your mouth	16.3	19.8	26.1	22.9	28.7
Found it uncomfortable to eat any foods	20.8	25.0	29.1	30.1	36.5
Been self‐conscious	22.5	26.4	44.6	38.6	40.9
Felt tense	13.6	17.0	28.5	30.1	28.7
Diet been unsatisfactory	5.9	6.5	16.4	11.1	19.1
Had to interrupt meals	5.5	7.1	15.5	9.8	23.5
Found it difficult to relax	9.4	11.0	21.9	13.7	22.6
Been a bit embarrassed	14.3	17.2	34.0	26.1	33.9
Been a bit irritable with other people	7.5	8.7	17.8	12.4	20.9
Had difficulty doing your usual jobs	3.6	3.6	8.5	3.9	16.5
Felt that life in general was less satisfying	10.0	12.2	21.0	19.0	19.1
Been totally unable to function	1.9	2.3	7.0	2.0	15.7

*Note:* ‘Yes’ indicates reporting an oral health impact on the OHIP item, defined as selecting Occasionally, Fairly often or Very often on the original five‐point response scale. Values represent the percentage of respondents within each smoking/vaping category who reported difficulty for each OHIP item.

#### 7.2.1 Prevalence of Oral Health Impacts (OHIP‐14) by Vaping History

Former e‐cigarette users who are ever smokers and current e‐cigarette users with smoking history generally show the highest prevalence across OHIP items. Patterns among current e‐cigarette users who have never smoked are variable and should be interpreted cautiously due to small subgroup size, reinforcing the importance of smoking history when interpreting associations involving vaping (see Table 3).

**TABLE 3:** Prevalence of oral health impacts (OHIP‐14) by vaping history.

OHIP item (any difficulty = Yes)	Never e‐cigarette users (%)	Former e‐cigarette users—all ever‐smokers (%)	Current e‐cigarette users—never smokers (%)	Current e‐cigarette users—ever smokers (%)
Had trouble pronouncing any words	4.2	10.3	7.7	7.5
Felt that your sense of taste has worsened	4.6	10.8	0.0	9.4
Had painful aching in your mouth	17.4	28.8	15.4	25.9
Found it uncomfortable to eat any foods	22.4	28.8	30.8	32.9
Been self‐conscious	24.0	45.3	23.1	40.4
Felt tense	14.8	29.5	23.1	29.8
Diet been unsatisfactory	6.3	14.4	7.7	14.9
Had to interrupt meals	6.4	12.7	7.7	16.1
Found it difficult to relax	10.1	22.1	15.4	17.6
Been a bit embarrassed	15.8	30.5	23.1	29.8
Been a bit irritable with other people	8.1	16.8	23.1	15.7
Had difficulty doing your usual jobs	3.7	7.9	15.4	9.0
Felt that life in general was less satisfying	10.9	19.9	23.1	18.8
Been totally unable to function	2.2	5.5	7.7	7.8

*Note:* ‘Yes’ indicates reporting an oral health impact on the OHIP item (Occasionally, Fairly often or Very often). Values represent the percentage of individuals within each vaping history category who reported the difficulty.

### 7.3 Crude and Adjusted Odds of Reporting Any Oral Health Difficulty

Table 4 presents the crude and adjusted associations between smoking/vaping status and the likelihood of reporting at least one OHIP‐14 oral health difficulty. Compared with never users, former smokers had modestly higher odds of reporting an oral health impact (adjusted OR = 1.25, 95% CI: 1.11–1.41). Current exclusive cigarette smokers had substantially elevated odds (adjusted OR = 1.85, 95% CI: 1.51–2.27), followed by exclusive e‐cigarette users (adjusted OR = 1.53, 95% CI: 1.10–2.13). Dual users also demonstrated significantly greater odds of reporting any oral health difficulty, although the magnitude was slightly lower than for exclusive smokers (adjusted OR = 1.55, 95% CI: 1.04–2.30). When vaping history was modelled separately, former e‐cigarette users (all of whom were ever smokers) had nearly twice the odds of reporting an oral health problem compared with those who had never used e‐cigarettes (adjusted OR = 1.98, 95% CI: 1.60–2.46). Current e‐cigarette users who had ever smoked also exhibited elevated odds (adjusted OR = 1.51, 95% CI: 1.16–1.97). Conversely, current e‐cigarette users who had never smoked showed lower odds of reporting oral health difficulties (adjusted OR = 0.52, 95% CI: 0.16–1.70), although the confidence interval was wide and non‐significant due to their very small sample size.

**TABLE 4:** Crude and adjusted odds ratios for reporting any oral health difficulty by current smoking/vaping status and combined vaping–smoking history.

Nicotine use group	Crude OR (95% CI), *p*	Adjusted OR (95% CI), *p*
Current smoking/vaping status		
Never users	1.00 (reference)	1.00 (reference)
Former smokers	1.22 (1.09–1.37), *p* = 0.001	1.25 (1.11–1.41), *p* < 0.001
Exclusive cigarette smokers	2.14 (1.76–2.61), *p* < 0.001	1.85 (1.51–2.27), *p* < 0.001
Exclusive e‐cigarette users	1.60 (1.16–2.21), *p* = 0.005	1.53 (1.10–2.13), *p* = 0.013
Dual users	1.93 (1.33–2.82), *p* = 0.001	1.55 (1.04–2.30), *p* = 0.030
Combined vaping–smoking history		
Never e‐cigarette users	1.00 (reference)	1.00 (reference)
Former e‐cigarette users (all ever‐smokers)	2.22 (1.81–2.74), *p* < 0.001	1.98 (1.60–2.46), *p* < 0.001
Current e‐cigarette users—never smokers	0.57 (0.18–1.87), *p* = 0.356	0.52 (0.16–1.70), *p* = 0.278
Current e‐cigarette users—ever smokers	1.70 (1.32–2.19), *p* < 0.001	1.51 (1.16–1.97), *p* = 0.002

*Note:* Any oral health difficulty was derived from 14 items of the Oral Health Impact Profile (OHIP‐14). Each item was originally measured on a five‐point scale (Never, Hardly ever, Occasionally, Fairly often, Very often). Responses of Occasionally, Fairly often or Very often were coded as ‘Yes’ (difficulty) and Never or Hardly ever as ‘No’. The composite outcome (AnyDiff) was coded as 1 if the participant reported difficulty on at least one OHIP item, and 0 otherwise. Crude odds ratios were obtained from unadjusted logistic regression. Adjusted odds ratios were obtained from logistic regression controlling for age group, sex, ethnicity, Index of Multiple Deprivation quintile, AUDIT‐C alcohol use, fluoride toothpaste use, ever use of smokeless tobacco, dental attendance frequency and brushing frequency.

### 7.4 Crude and Adjusted Ordered Logistic Regression for Number of OHIP Problems

Table 5 summarises the graded association between smoking/vaping status and the number of reported oral health impacts. Relative to never users, former smokers demonstrated significantly higher odds of reporting multiple OHIP‐14 problems (adjusted OR = 1.28, 95% CI: 1.15–1.44). The strongest associations were observed among exclusive cigarette smokers (adjusted OR = 1.99, 95% CI: 1.65–2.39) and dual users (adjusted OR = 1.89, 95% CI: 1.31–2.73). Exclusive e‐cigarette users also showed increased odds of reporting more oral health problems compared with never users (adjusted OR = 1.79, 95% CI: 1.31–2.44). Analyses using combined vaping–smoking history revealed similar patterns. Former e‐cigarette users (all of whom were ever smokers) had almost twice the odds of reporting higher OHIP burden versus those who had never vaped (adjusted OR = 1.90, 95% CI: 1.57–2.29). Current e‐cigarette users who were ever smokers also showed significantly elevated odds (adjusted OR = 1.74, 95% CI: 1.36–2.22). Conversely, current e‐cigarette users who had never smoked appeared less likely to report multiple OHIP problems (adjusted OR = 0.69, 95% CI: 0.21–2.30), although this estimate was not statistically significant, reflecting sparse numbers in this group and limited statistical precision.

**TABLE 5:** Crude and adjusted ordered logistic regression for number of OHIP problems.

Nicotine use group	Crude OR (95% CI), *p*	Adjusted OR (95% CI), *p*
Current smoking/vaping status		
Never users	1.00 (reference)	1.00 (reference)
Former smokers	1.24 (1.11–1.38), *p* < 0.001	1.28 (1.15–1.44), *p* < 0.001
Exclusive cigarette smokers	2.32 (1.94–2.78), *p* < 0.001	1.99 (1.65–2.39), *p* < 0.001
Exclusive e‐cigarette users	1.83 (1.35–2.48), *p* < 0.001	1.79 (1.31–2.44), *p* < 0.001
Dual users	2.49 (1.75–3.54), *p* < 0.001	1.89 (1.31–2.73), *p* = 0.001
Combined vaping–smoking history		
Never e‐cigarette users	1.00 (reference)	1.00 (reference)
Former e‐cigarette users (all ever‐smokers)	2.16 (1.80–2.58), *p* < 0.001	1.90 (1.57–2.29), *p* < 0.001
Current e‐cigarette users—never smokers	0.78 (0.23–2.60), *p* = 0.682	0.69 (0.21–2.30), *p* = 0.545
Current e‐cigarette users—ever smokers	1.99 (1.57–2.52), *p* < 0.001	1.74 (1.36–2.22), *p* < 0.001

*Note:* Odds ratios are derived from ordered logistic regression models with Never users or Never e‐cigarette users as the reference category. Crude estimates are unadjusted. Adjusted models control for age group, sex, ethnicity, Index of Multiple Deprivation quintile, alcohol consumption (AUDIT‐C), fluoride toothpaste use, ever use of smokeless tobacco, dental attendance frequency and brushing frequency. The outcome NumOHIPg is a four‐level ordinal measure summarising the number of OHIP‐14 items with reported impact: 0 = none, 1 = one item, 2 = 2–4 items, 3 = five or more items. Higher odds ratios indicate greater likelihood of reporting more oral health problems.

### 7.5 Adjusted Associations Between Smoking/Vaping Status and Specific Oral Health Outcomes

Table 6 presents fully adjusted odds ratios for three self‐reported oral health indicators, loose teeth, gum disease history and dry mouth, across current smoking and vaping categories. Relative to never users, former smokers had significantly elevated odds of loose teeth (adjusted OR = 1.80, 95% CI: 1.52–2.13), gum disease history (adjusted OR = 1.39, 95% CI: 1.20–1.61) and dry mouth (adjusted OR = 1.24, 95% CI: 1.10–1.39). Stronger associations were observed among exclusive cigarette smokers, who had over three times the odds of loose teeth (adjusted OR = 3.18, 95% CI: 2.47–4.09), with almost double the odds of gum disease history (adjusted OR = 1.94, 95% CI: 1.52–2.49) and 70% higher odds of dry mouth (adjusted OR = 1.70, 95% CI: 1.39–2.08). Exclusive e‐cigarette users also exhibited increased odds compared with never users, including more than double the odds of loose teeth (adjusted OR = 2.42, 95% CI: 1.58–3.70) and gum disease history (adjusted OR = 2.16, 95% CI: 1.47–3.15) and 69% higher odds of dry mouth (adjusted OR = 1.69, 95% CI: 1.22–2.34). Dual users also showed among the strongest associations, with over threefold increased odds of loose teeth (adjusted OR = 3.11, 95% CI: 1.95–4.97) and the highest odds of gum disease history (adjusted OR = 3.21, 95% CI: 2.06–4.99), alongside significantly elevated reports of dry mouth (adjusted OR = 1.52, 95% CI: 1.03–2.26).

**TABLE 6:** Adjusted associations between smoking/vaping status and oral health outcomes (loose teeth, gum disease history and dry mouth).

Nicotine use group	Loose teeth—adjusted OR (95% CI), *p*	Gum disease—adjusted OR (95% CI), *p*	Dry mouth—adjusted OR (95% CI), *p*
Current smoking/vaping status			
Never users	1.00 (reference)	1.00 (reference)	1.00 (reference)
Former smokers	1.80 (1.52–2.13), *p* < 0.001	1.39 (1.20–1.61), *p* < 0.001	1.24 (1.10–1.39), *p* < 0.001
Exclusive cigarette smokers	3.18 (2.47–4.09), *p* < 0.001	1.94 (1.52–2.49), *p* < 0.001	1.70 (1.39–2.08), *p* < 0.001
Exclusive e‐cigarette users	2.42 (1.58–3.70), *p* < 0.001	2.16 (1.47–3.15), *p* < 0.001	1.69 (1.22–2.34), *p* = 0.001
Dual users	3.11 (1.95–4.97), *p* < 0.001	3.21 (2.06–4.99), *p* < 0.001	1.52 (1.03–2.26), *p* = 0.036
Combined vaping–smoking history			
Never e‐cigarette users	1.00 (reference)	1.00 (reference)	1.00 (reference)
Former e‐cigarette users (all ever‐smokers)	1.98 (1.51–2.59), *p* < 0.001	2.01 (1.57–2.59), *p* < 0.001	1.41 (1.15–1.73), *p* = 0.001
Current e‐cigarette users—never smokers	0.52 (0.07–4.14), *p* = 0.540	1.06 (0.23–5.03), *p* = 0.937	0.26 (0.08–0.84), *p* = 0.025
Current e‐cigarette users—ever smokers	2.07 (1.50–2.85), *p* < 0.001	2.32 (1.73–3.13), *p* < 0.001	1.60 (1.24–2.08), *p* < 0.001

*Note:* Adjusted odds ratios are derived from multivariable models controlling for age group, sex, ethnicity, Index of Multiple Deprivation quintile, alcohol consumption (AUDIT‐C), fluoride toothpaste use, ever use of smokeless tobacco, dental attendance frequency and brushing frequency. Loose teeth and gum disease history were modelled using logistic regression. Dry mouth was modelled using ordered logistic regression.

Patterns were largely consistent when exposures were re‐classified using combined vaping–smoking history. Former e‐cigarette users (all of whom were ever smokers) had nearly double the odds of loose teeth (adjusted OR = 1.98, 95% CI: 1.51–2.59) and gum disease history (adjusted OR = 2.01, 95% CI: 1.57–2.59) and were significantly more likely to report dry mouth (adjusted OR = 1.41, 95% CI: 1.15–1.73). Current e‐cigarette users who had never smoked showed no statistically significant excess odds for loose teeth or gum disease history, although they appeared less likely to report dry mouth (adjusted OR = 0.26, 95% CI: 0.08–0.84). In contrast, current e‐cigarette users with a smoking history had significantly elevated odds of all three outcomes, including loose teeth (adjusted OR = 2.07, 95% CI: 1.50–2.85), gum disease history (adjusted OR = 2.32, 95% CI: 1.73–3.13) and dry mouth (adjusted OR = 1.60, 95% CI: 1.24–2.08).

### 7.6 Associations Between Smoking/e‐Cigarette Status and Self‐Rated Dental Health

Table 7 presents the adjusted associations between smoking/e‐cigarette status and self‐rated dental health. Compared with never users, all nicotine exposure groups reported significantly poorer dental health. Former smokers had 44% higher odds of poorer self‐rated dental health (adjusted OR = 1.44, 95% CI: 1.26–1.63). The strongest associations were observed among exclusive cigarette smokers, who were almost three times more likely to report poorer dental health than never users (adjusted OR = 2.88, 95% CI: 2.36–3.52). Exclusive e‐cigarette users also demonstrated substantially worse perceived dental health, with more than double the odds compared with never users (adjusted OR = 2.45, 95% CI: 1.77–3.39). Similarly, dual users exhibited significantly elevated odds of poorer dental health relative to never users (adjusted OR = 2.45, 95% CI: 1.68–3.58).

**TABLE 7:** Adjusted associations between smoking/e‐cigarette status and self‐rated dental health.

Nicotine use group	Adjusted OR for poorer dental health (95% CI), *p*
Current smoking/vaping status	
Never users	1.00 (reference)
Former smokers	1.44 (1.26–1.63), *p* < 0.001
Exclusive cigarette smokers	2.88 (2.36–3.52), *p* < 0.001
Exclusive e‐cigarette users	2.45 (1.77–3.39), *p* < 0.001
Dual users	2.45 (1.68–3.58), *p* < 0.001
Combined vaping–smoking history	
Never e‐cigarette users	1.00 (reference)
Former e‐cigarette users (all ever‐smokers)	2.24 (1.82–2.77), *p* < 0.001
Current e‐cigarette users—never smokers	0.97 (0.32–2.94), *p* = 0.952
Current e‐cigarette users—ever smokers	2.16 (1.67–2.79), *p* < 0.001

*Note:* Adjusted odds ratios derived from ordered logistic regression. Higher values indicate greater odds of reporting poorer dental health (Very good/good, Fair and Bad/very bad). Models adjusted for age group, sex, ethnicity, Index of Multiple Deprivation quintile, AUDIT‐C alcohol use, fluoride toothpaste use, smokeless tobacco use, dental attendance frequency and brushing frequency.

This pattern was consistent when vaping–smoking history was analysed separately. Former e‐cigarette users (all ever‐smokers) had more than twice the odds of poorer dental health compared with never e‐cigarette users (adjusted OR = 2.24, 95% CI: 1.82–2.77), while current e‐cigarette users who had smoked also reported poorer dental health (adjusted OR = 2.16, 95% CI: 1.67–2.79). In contrast, current e‐cigarette users who had never smoked did not differ from never users (adjusted OR = 0.97, 95% CI: 0.32–2.94), although the wide confidence interval reflects small sample size.

### 7.7 Sensitivity Analyses

Sensitivity analyses additionally adjusted the fully adjusted models for denture status, number of natural teeth and self‐rated general health. Overall, the pattern of results was broadly similar to the primary adjusted analyses, although several estimates were attenuated. In the current smoking/vaping status models, the direction of the associations was preserved across all outcomes, with some attenuation—particularly for the binary ‘any oral health difficulty’ outcome among exclusive e‐cigarette users and dual users, where confidence intervals crossed the null, while estimates for exclusive cigarette smokers, dual users and former smokers remained statistically significant for loose teeth, gum disease history, OHIP burden and self‐rated dental health. In the vaping–smoking history models, elevated odds remained concentrated among participants with a history of cigarette smoking: former e‐cigarette users (all ever‐smokers) and current e‐cigarette users with a smoking history retained statistically significant elevated odds across all six outcomes, while estimates for the very small subgroup of current e‐cigarette users who had never smoked (*n* = 13) remained imprecise, with wide confidence intervals that should be interpreted as exploratory rather than as evidence of no association. Full results are presented in Table S1.

Firth penalised logistic regression, conducted as a sparse‐data sensitivity analysis for the binary outcomes, produced conclusions consistent with the main models: elevated odds among former e‐cigarette users and current e‐cigarette users with a smoking history were preserved, while estimates for never‐smoking current e‐cigarette users remained too imprecise for firm conclusions. Detailed Firth estimates are provided in Table S2.

## 8 Discussion

This study provides new evidence on the oral health burden associated with conventional cigarette smoking, e‐cigarette use and dual use within a nationally representative adult population in England. A clear pattern of association emerged, whereby exclusive cigarette smokers and dual users generally showed the highest prevalence and odds of adverse oral health outcomes across OHIP‐14 domains, including functional limitation, pain, psychological discomfort and social restriction. Former smokers also demonstrated elevated odds of oral health impacts, consistent with the possibility that smoking‐related oral damage may persist after cessation. These findings align with established literature showing that smoking is associated with impaired periodontal integrity, reduced soft‐tissue perfusion, altered salivary function and poorer oral health‐related quality of life (Schwarz et al. 2023; Arruda et al. 2024; Rad et al. 2010).

Exclusive e‐cigarette users also displayed higher odds of several adverse self‐reported oral health outcomes compared with never users, although the magnitudes were generally smaller than those observed among exclusive cigarette smokers and dual users. These associations are descriptive and cannot, on their own, indicate that e‐cigarette use directly contributes to oral health burden, because interpretation is complicated by prior smoking history and by the absence of detailed exposure information. In the primary current‐use models, the exclusive e‐cigarette category included both never smokers who currently vaped and former smokers who had switched to e‐cigarettes. Therefore, elevated odds in this group may partly reflect cumulative or residual oral health consequences of previous combustible cigarette smoking rather than e‐cigarette use alone, and the absence of information on smoking intensity, duration, pack‐years, time since cessation, e‐cigarette frequency, vaping duration, device type, nicotine concentration and product composition prevents these residual contributions from being quantified. This interpretation is consistent with evidence from NHANES 2015–2018 suggesting that associations between e‐cigarette use and self‐reported periodontal disease were more apparent among people with current or previous smoking exposure than among never smokers (AlQobaly et al. 2022). Recent reviews have also reported that e‐cigarette use may be associated with plaque accumulation, inflammatory changes and oral microbiome alterations, but that observed periodontal changes are generally smaller than those associated with cigarette smoking (Charde et al. 2024; Tattar et al. 2025). Given variation in exposure definitions, device types, product composition and study design across the literature, these findings require cautious interpretation.

The combined vaping–smoking history models provided important clarification. Former e‐cigarette users, all of whom had previously smoked, showed elevated odds of adverse self‐reported oral health outcomes, consistent with the possibility that prior combustible tobacco exposure may explain part of the observed burden in people with a history of vaping. Current e‐cigarette users with a smoking history also had higher odds of several outcomes, including loose teeth, gum disease history, dry mouth, OHIP burden and poorer self‐rated dental health. However, because the dataset did not capture smoking intensity, duration, pack‐years, time since cessation or cumulative vaping exposure, the relative contributions of past combustible smoking and current e‐cigarette use cannot be disentangled in these models. Findings for current e‐cigarette users who had never smoked are exploratory. This subgroup was extremely small (*n* = 13), and the wide confidence intervals across outcomes reflect limited statistical precision rather than a reliable null effect. The lower odds observed for dry mouth in this subgroup, in particular, should not be read as protective and may reflect sparse data, residual confounding, reporting variation or chance.

Dual users remained an important high‐burden group. They showed the highest odds of gum disease history across all groups (adjusted OR = 3.21), with odds comparable to exclusive cigarette smokers for loose teeth and OHIP burden, although their odds for any oral health difficulty, dry mouth and self‐rated dental health were lower than those of exclusive cigarette smokers. This pattern may reflect accumulated exposure to combustible tobacco, concurrent nicotine product use, heavier dependence, poorer oral health behaviours, delayed dental attendance or other unmeasured behavioural and health‐related factors. Persistently elevated odds among former smokers also suggest that cumulative tobacco exposure may have lasting relevance for oral health, including periodontal damage and tooth loss (Aldalaeen et al. 2025; Thiem et al. 2023; Duarte et al. 2022; ALHarthi et al. 2019; Souto et al. 2019).

The sensitivity analyses (Tables S1 and S2) supported the direction and broad consistency of the main findings, with some attenuation. After additional adjustment for denture status, number of natural teeth and self‐rated general health, exclusive cigarette smoking remained associated with higher odds across all outcomes, and dual use remained associated with loose teeth, gum disease history, greater OHIP burden and poorer self‐rated dental health, while associations for any oral health difficulty among exclusive e‐cigarette users and dual users were attenuated to non‐significance. This attenuation was expected because denture wearing and number of natural teeth may partly capture accumulated oral disease burden and tooth loss, which may lie downstream of long‐term smoking exposure. In the vaping–smoking history models, elevated odds remained concentrated among former e‐cigarette users and current e‐cigarette users with a smoking history. Firth penalised logistic regression further supported this interpretation, with estimates for current e‐cigarette users who had never smoked remaining imprecise and exploratory.

This study also contributes to the evidence base by demonstrating parallel gradients in self‐rated dental health. Poorer self‐rated dental health was more likely among exclusive cigarette smokers, exclusive e‐cigarette users and dual users, with former smokers also showing elevated odds. Current e‐cigarette users without smoking history did not differ meaningfully from never e‐cigarette users, although interpretation remains limited by the small subgroup size. These findings provide convergence between OHIP‐14 impacts, self‐reported oral health indicators and broader self‐perceived dental health. However, self‐rated dental health is shaped not only by disease status but also by oral health literacy, expectations, awareness of symptoms, access to dental care and previous experiences with dental services. This is important because participants with similar oral conditions may rate their oral health differently depending on knowledge, expectations and care‐seeking behaviour.

Because the present analysis is cross‐sectional and based on self‐reported, nationally representative survey data from English adults, any broader clinical or public health implications should be regarded as hypothesis‐generating rather than definitive, and generalisability is limited to comparable survey populations. Within these constraints, the findings reaffirm that combustible cigarette smoking is consistently and strongly associated with poorer self‐reported oral health, supporting the continued integration of smoking cessation advice within dental and primary care settings. They also suggest that future studies of e‐cigarette use and oral health should distinguish between never smokers, former smokers, current smokers and dual users, because combining these groups can obscure the contribution of prior combustible tobacco exposure. The elevated odds observed among exclusive e‐cigarette users and dual users do not, on their own, establish that e‐cigarettes directly cause oral health harm, but they argue against presenting these products as neutral with respect to oral health pending evidence from longitudinal studies with detailed exposure data. The estimates among never‐smoking current e‐cigarette users were too sparse to support any conclusion about oral health outcomes in nicotine‐naïve users. Dental practitioners may nevertheless find it useful to record both current nicotine product use and smoking history when assessing oral health risk and providing smoking cessation advice (Kumar et al. 2026).

This study has several strengths, including the use of a large nationally representative survey, simultaneous modelling of current and historical smoking‐vaping exposure, adjustment for sociodemographic, behavioural, oral hygiene and dental attendance factors, and the inclusion of multiple patient‐reported oral health outcomes. Additional sensitivity analyses accounting for denture status, number of natural teeth and general health status further tested the robustness of the findings.

However, several limitations should be acknowledged. The cross‐sectional design precludes causal or temporal inference, and the observed associations cannot establish whether smoking or vaping preceded the reported oral health outcomes, nor can dose–response patterns or independent effects of e‐cigarette use apart from prior smoking exposure be inferred from these data. The exposure and outcome measures were self‐reported, and loose teeth, gum disease history, dry mouth and self‐rated dental health were not clinically verified; these are relevant patient‐reported indicators but are not equivalent to clinically confirmed periodontal or oral disease diagnoses, and the absence of clinical examination, objective measures of oral dryness and validated periodontal endpoints limits diagnostic certainty. The dataset did not include detailed measures of cigarette smoking intensity, duration, pack‐years, age at initiation, time since cessation, e‐cigarette frequency, duration of vaping, device type, nicotine concentration or product composition, and elevated odds among former smokers, exclusive e‐cigarette users and dual users may therefore partly reflect cumulative or residual effects of previous combustible cigarette exposure that cannot be quantified in the present analysis.

Specific chronic diseases, such as diabetes and hypertension, and medication use affecting salivary flow were also unavailable, although self‐rated general health was included as a broad proxy in sensitivity analyses. Restricting the analytic sample to individuals who completed the survey may have introduced selection bias, as respondents with better general and oral health may have been more willing or able to participate, which could partly explain the relatively low prevalence of OHIP‐14 impacts observed in this sample and limits generalisability to the wider English adult population and to comparable survey populations. Finally, the subgroup of current e‐cigarette users who had never smoked was very small, limiting precision and preventing firm conclusions for this group; results for this subgroup are exploratory and the wide confidence intervals should not be interpreted as evidence of no effect. Future longitudinal studies with detailed smoking and vaping histories, clinical periodontal assessments, objective measures of oral dryness where relevant, clinically validated oral health endpoints, medication data and biomarker‐validated exposure measures are needed to clarify temporal relationships and product‐specific oral health outcomes.

## 9 Conclusion

In this cross‐sectional, population‐based analysis of English adults, combustible cigarette smoking and dual use were associated with poorer self‐reported oral health outcomes, with former smokers also showing elevated odds compared with never users. Findings for exclusive e‐cigarette users require cautious interpretation because most had a prior smoking history, while results for never‐smoking current e‐cigarette users remain exploratory. Smoking cessation should therefore remain central to oral disease prevention, and dental practitioners may find it useful to record both current nicotine product use and smoking history when assessing oral health risk. Longitudinal studies with detailed exposure histories and clinically validated oral health endpoints are needed to clarify temporal and product‐specific associations.

## Author Contributions

**Yusuff Adebayo Adebisi:** conceptualization, methodology, software, formal analysis, data curation, supervision, investigation, writing – original draft, writing – review and editing. **Waleed Almutairi:** writing – review and editing, investigation, validation, methodology. **Najim Z. Alshahrani:** methodology, validation, data curation, supervision, writing – review and editing. **Isaac Olushola Ogunkola:** writing – review and editing, data curation, investigation, validation, methodology. **Roqayya Mohammed Ahmed Alhayyani:** writing – review and editing, project administration, validation. **Oshibe Joseph Daberechi:** writing – review and editing, methodology, validation, investigation. **Mona Majrashi:** writing – review and editing, investigation, validation, methodology. **Anas Ali Alhur:** writing – review and editing, resources, supervision, methodology. **Mohammed Moshabbab Alqahtani:** writing – review and editing, investigation, validation, methodology, software.

## Funding

The authors have nothing to report. Open‐access publication fees were supported by the University of Glasgow through institutional agreements.

## Ethics Statement

This study used anonymised, publicly available secondary data. As the analysis did not involve identifiable human subjects, no ethical approval or informed consent was required, and the research qualifies for exemption from human subjects review.

## Conflicts of Interest

The authors declare no conflicts of interest. Y.A.A. has previously received funding through the Tobacco Harm Reduction Scholarship and the Kevin Molloy Fellowship, both awarded by Knowledge‐Action‐Change (KAC), an independent public health organisation based in the UK. KAC is funded by Global Action to End Smoking (GA).

## Supporting information


**Table S1:** Sensitivity analyses: adjusted odds ratios for oral health outcomes after additional adjustment for denture status, number of natural teeth and self‐rated general health.
**Table S2:** Firth penalised logistic regression: sparse‐data sensitivity analysis for binary outcomes.

## Data Availability

The dataset analysed in this study is publicly accessible. Data can be downloaded from the UK Data Service repository.
