# Supplementary material for: Heterogeneous Oral Health Outcomes by Combustible Cigarette Smoking, E‐Cigarette Use and Dual Use: A Cross‐Sectional Population‐Based Analysis
Source: Oral Dis. 2026 Jun 12;32(10):3343–55. doi: 10.1111/odi.70390 (PMC13768904; doi:10.1111/odi.70390)
Supplement: Supplementary file 1 [file ODI-32-3343-s001.docx]

# **Supplementary Material**

Heterogeneous Oral Health Outcomes by Combustible Cigarette Smoking, E-Cigarette Use, and Dual Use: A Cross-Sectional Population-Based Analysis

This supplementary file presents the detailed results of the sensitivity analyses described in the main manuscript. Supplementary Table S1 reports adjusted odds ratios for the six oral health outcomes after additional adjustment for denture status, number of natural teeth, and self-rated general health, on top of the covariates used in the primary models. Supplementary Table S2 reports Firth penalised logistic regression estimates for the three binary outcomes, conducted as a sparse-data sensitivity analysis to address the very small subgroup of current e-cigarette users who had never smoked.

**Supplementary Table S1. Sensitivity analyses: adjusted odds ratios for oral health outcomes after additional adjustment for denture status, number of natural teeth, and self-rated general health**

| **Nicotine use group** | **Any oral health difficulty OR (95% CI)** | **Loose teeth OR (95% CI)** | **Gum disease history OR (95% CI)** | **Number of OHIP impacts OR (95% CI)** | **Dry mouth OR (95% CI)** | **Self-rated dental health OR (95% CI)** |
| --- | --- | --- | --- | --- | --- | --- |
| **Current smoking / vaping status** | | | | | | |
| Never users | 1.00 (reference) | 1.00 (reference) | 1.00 (reference) | 1.00 (reference) | 1.00 (reference) | 1.00 (reference) |
| Former smokers | 1.15 (1.02–1.30) | 1.57 (1.31–1.87) | 1.33 (1.15–1.54) | 1.18 (1.05–1.32) | 1.16 (1.03–1.30) | 1.26 (1.11–1.45) |
| Exclusive cigarette smokers | 1.58 (1.28–1.95) | 2.73 (2.08–3.58) | 1.81 (1.40–2.34) | 1.66 (1.38–2.01) | 1.45 (1.18–1.77) | 2.21 (1.79–2.73) |
| Exclusive e-cigarette users | 1.35 (0.96–1.89) | 1.94 (1.24–3.04) | 2.03 (1.38–2.97) | 1.62 (1.18–2.21) | 1.50 (1.09–2.08) | 2.05 (1.46–2.86) |
| Dual users | 1.34 (0.89–1.99) | 2.55 (1.54–4.22) | 3.17 (2.02–5.00) | 1.62 (1.12–2.34) | 1.28 (0.86–1.89) | 2.00 (1.35–2.96) |
| **Combined vaping–smoking history** | | | | | | |
| Never e-cigarette users | 1.00 (reference) | 1.00 (reference) | 1.00 (reference) | 1.00 (reference) | 1.00 (reference) | 1.00 (reference) |
| Former e-cigarette users (all ever-smokers) | 1.85 (1.48–2.31) | 1.81 (1.36–2.41) | 1.92 (1.48–2.48) | 1.77 (1.46–2.14) | 1.29 (1.05–1.59) | 1.94 (1.56–2.41) |
| Current e-cigarette users — never smokers (n = 13)* | 0.50 (0.15–1.71) | 0.49 (0.06–4.13) | 1.05 (0.21–5.11) | 0.71 (0.22–2.36) | 0.26 (0.08–0.86) | 0.92 (0.29–2.89) |
| Current e-cigarette users — ever smokers | 1.36 (1.04–1.78) | 1.77 (1.25–2.49) | 2.25 (1.67–3.04) | 1.60 (1.25–2.04) | 1.42 (1.10–1.85) | 1.89 (1.45–2.46) |

Adjusted odds ratios are derived from logistic regression (any oral health difficulty, loose teeth, gum disease history) and ordered logistic regression (number of OHIP impacts, dry mouth, self-rated dental health). All models additionally adjusted for age group, sex, ethnicity, Index of Multiple Deprivation quintile, alcohol consumption (AUDIT-C), fluoride toothpaste use, ever use of smokeless tobacco, dental attendance frequency, and brushing frequency, together with denture status, number of natural teeth, and self-rated general health. For loose teeth and gum disease history models including dentition status, participants with no natural teeth were omitted because edentulous status perfectly predicted absence or non-applicability of the outcome. *Estimates for current e-cigarette users who had never smoked are exploratory and should not be interpreted as evidence of absence of association; wide confidence intervals reflect limited statistical precision rather than a reliable null effect.

**Supplementary Table S2. Firth penalised logistic regression: sparse-data sensitivity analysis for binary outcomes**

| **Nicotine use group** | **Any oral health difficulty Firth OR (95% CI)** | **Loose teeth Firth OR (95% CI)** | **Gum disease history Firth OR (95% CI)** |
| --- | --- | --- | --- |
| **Combined vaping–smoking history** | | | |
| Never e-cigarette users | 1.00 (reference) | 1.00 (reference) | 1.00 (reference) |
| Former e-cigarette users (all ever-smokers) | 1.97 (1.59–2.45) | 1.99 (1.51–2.60) | 2.03 (1.58–2.62) |
| Current e-cigarette users — never smokers (n = 13)* | 0.55 (0.18–1.72) | 0.74 (0.13–4.15) | 1.26 (0.30–5.18) |
| Current e-cigarette users — ever smokers | 1.51 (1.16–1.96) | 2.04 (1.48–2.82) | 2.32 (1.72–3.11) |

Firth penalised logistic regression was conducted as a sparse-data sensitivity analysis for the binary outcomes given the very small subgroup of current e-cigarette users who had never smoked. Models adjusted for the same covariates as in the primary analyses (age group, sex, ethnicity, Index of Multiple Deprivation quintile, alcohol consumption [AUDIT-C], fluoride toothpaste use, ever use of smokeless tobacco, dental attendance frequency, and brushing frequency). Firth estimates were consistent with the main logistic regression analyses, with elevated odds among former e-cigarette users and current e-cigarette users with a smoking history. *Estimates for current e-cigarette users who had never smoked remained imprecise and should be interpreted as exploratory.
